# The Use of Intuition in Homeopathic Clinical Decision Making: An Interpretative Phenomenological Study

**DOI:** 10.1093/ecam/nep153

**Published:** 2011-01-12

**Authors:** Sarah Brien, Bridget Dibb, Alex Burch

**Affiliations:** ^1^Department of Primary Care, University of Southampton, Southampton, Hampshire SO16 5ST, UK; ^2^School of Social Sciences, Brunel University, Uxbridge, UK

## Abstract

While intuition plays a role in clinical decision making within conventional medicine, little is understood about its use in complementary and alternative medicine (CAM). The aim of this qualitative study was to investigate intuition from the perspective of homeopathic practitioners; its' manifestation, how it was recognized, its origins and when it was used within daily clinical practice. Semi-structured interviews were carried out with clinically experienced non-National Health Service (NHS) UK homeopathic practitioners. Interpretative phenomenological analysis was used to analyze the data. Homeopaths reported many similarities with conventional medical practitioner regarding the nature, perceived origin and manifestation of their intuitions in clinical practice. Intuition was used in two key aspects of the consultation: (i) to enhance the practitioner-patient relationship, these were generally trusted; and (ii) intuitions relating to the prescribing decision. Homeopaths were cautious about these latter intuitions, testing any intuitive thoughts through deductive reasoning before accepting them. Their reluctance is not surprising given the consequences for patient care, but we propose this also reflects homeopaths' sensitivity to the academic and medical mistrust of both homeopathy and intuition. This study is the first to explore the use of intuition in decision making in any form of complementary medicine. The similarities with conventional practitioners may provide confidence in validating intuition as a legitimate part of the decision making process for these specific practitioners. Further work is needed to elucidate if these findings reflect intuitive use in clinical practice of other CAM practitioners in both private and NHS (i.e., time limited) settings.

## 1. Introduction

The use of evidence-based knowledge in clinical decision making has been widely explored but the role of intuition has also emerged as important [1–7]. Using intuition as a “clinical tool” [8] in decision making has to date, been explored mainly in nursing populations [1, 4], but also in occupational medicine and general practitioner consultations [8, 9]. There is poor consensus concerning its definition [10, 11], and 74 definitions have been described [12] suggesting it means different things to different people [13]. There are also conflicting opinions as to whether or not it is based on logical, reasoned thought [3, 10, 14, 15]. There are some salient features common to many definitions, including, a characterization of its speed [1, 16, 17] and the difficulty individuals have in explaining how they came to an intuitive decision [18]. It has been described as a multi-dimensional concept synonymous with insight and instinct, an understanding based on background knowledge, a skill developed through previous experiential knowledge, as well as a connection between patients and practitioners [3, 12].

Key aspects of intuition have been identified in clinical practice (e.g., [1]) using Dreyfus's criteria [19]: some relating specifically to perception, that is, pattern recognition, similarity recognition and sense of salience; and others include common sense understanding, skilled know how, deliberate rationality and devaluing intuitive judgment. Some researchers [20–22] categorize intuition into three types: cognitive inference, gestalt intuition and precognitive functioning. In addition, intuition is proposed as being used for one of six functions: discovery (“the flashbulb moment”), creativity (generating alternative options), evaluation (aid a final decision, a “yes or no” signal), operation (“luck”), prediction (ability to predict future events) and illumination (nirvana, a spiritual level of awareness) [20].

Only a few studies have explicitly focused on the nature and role of intuition in clinical practice [17, 22–24]. Specifically, Rew [17] identified that nurses use intuition during assessment, implementation and/or intervention stages of a therapeutic consultation but would not respond to their intuitions when it came to planning, diagnosis, or evaluation. Response to intuitions were shown to be manifested through affective behaviors (e.g., nurse were afraid to leave their patient); in a cognitive way (e.g., they would seek out more information); and through a four step behavioral response, that is, they would seek additional data, then attempt to corroborate their intuitions, report their findings to the doctor directly or on the patient notes, and finally perform a specific intervention.

Our understanding of intuition in conventional medical practitioners' decision making is still limited. Benner's [25] seminal work, supported by subsequent studies [10, 24, 26–29], reported that the use of intuition distinguished expert from non-expert professionals. However, previous life-related intuitive experiences [12] also aid intuition in clinical practice. Intuition is used in situations where communication is ambiguous or indirect [29], in uncertain or complex situations [30] especially where a practitioner needs to think on their feet because there is no time for slower rational thinking [31]. It is also used to encourage a holistic approach [32] and to facilitate connecting with a patient to improve decision making and patient outcomes [33].

Our understanding of intuition in the complementary and alternative medicine (CAM) practitioner's decision making is, by comparison, even more limited. However, with the escalating use of CAM [34], it is important to understand how CAM practitioners make their decisions. Intuition may be used and viewed differently from conventional medical practitioners due to differences in their consultations and their paradigm. CAM consultations are more holistic, patient centered and collaborative [35] than conventional medicine consultations, so consequently practitioners may be more perceptive of sensory and psycho-social information. In addition, the length and nature of the consultations can be different; for example, homeopathic consultations can last up to one and a half hours for new patients and involve a very full exploration of the patient's narrative of their illness and symptoms [36]. Through this lengthy consultation process classically trained homeopaths select one out of approximately four thousand remedies to prescribe for their patient; this decision process is therefore complex and dependent on a deep understanding of the patient.

Two studies have assessed intuition in CAM to date. Brien et al. [37] asked experienced homeopathic clinicians to rate paper case reports (on a scale of 1–10) to identify the extent to which they used both clinical facts and their intuition, when making a decision as to whether individuals had responded to a homeopathic remedy. All homeopaths used factual evidence as well as intuition, but they relied more on intuition when deciding if the individual had responded to the medication. However, the methodological limitations of this exploratory article may not be representative of how homeopaths prescribe in clinical practice [38]. The results of this study led to the development of another study [2] of which the current study forms a part, which qualitatively explored decision making processes in homeopaths. The findings resulted in the development of a decision making model (PHIRM) which demonstrates the use of intuition, amongst other processes by homeopathic practitioners. However, as highlighted by Brien et al. [37], understanding what intuition *means* to homeopathic practitioners still remains unexplored. As intuition has been viewed by some with skepticism we were interested to see whether homeopathy, a medical approach not new to skepticism [39], used intuition to inform clinical decision making. The aim of this qualitative study was therefore to explore what intuition meant to homeopathic practitioners and how it arose within their daily clinical practice.

## 2. Methods

In-depth, face-to-face, semi structured interviews were carried out with non-National Health Service (NHS) homeopathic practitioners who were members of the Society of Homeopaths. Participants were recruited through 19 private CAM clinics based in Southern England during February to December 2006. They were contacted by telephone to ensure eligibility (current conduct of weekly clinical practice, and more than 3 years clinical homeopathic experience). Participants were then selected by purposive sampling in order to obtain a wide-ranging sample (across clinical experience, gender and geographical location). Written informed consent was obtained prior to commencement of interviews and participants were informed of their right to withdraw at any time.

Interviews took place at practitioners' private practices and lasted *∼*60 min. Interviews followed a narrative style commencing with an open ended question asking participants to talk about their decision making processes particularly focusing on their first consultation with a patient. The role of intuition was subsequently explored within the frame of decision making, specifically what intuition meant to them, how they would define it and when they would use it. Interviews were audio-taped and transcribed verbatim, using pseudonyms to protect anonymity. Ethical approval was sought and received from the Ethics Committee at the Host institution.

### 2.1. Data Analysis

The data was analyzed using Interpretative Phenomenological Analysis (IPA [40]) resulting in the development of themes within and across interviews. IPA allows an examination of the participants' perceptions while recognizing the researchers' influence and interpretation. It is an idiographic approach involving detailed analysis of each case to produce a thick interpretative account of a smaller number of participants, rather than a thinner account of a larger sample. Four themes emerged inductively from the data and are presented below. Regular meetings by all the authors ensure that a reflexive approach was taken regarding the analysis and interpretation of the data. At the meetings the team reviewed and revised the emerging categories and subsequent conceptual models. This iterative process was aided by discussion and reflective feedback to identify biases, overstatements and discrepancies in the analytical and interpretative phases to ensure the final model presented was fully supported by the data.

## 3. Results

### 3.1. Sample Characteristics

Of the 32 homeopaths contacted, 15 agreed to take part, all practiced classical (individualized) homeopathy; 14 were interviewed as one participant withdrew from the study. Five were male, nine were female. They were all non-medical private practitioners based in south of England working in a mix of rural, town and inner city areas, ranging in length of practice from 6 to 25 years.

### 3.2. Themes

**Figure 1:**
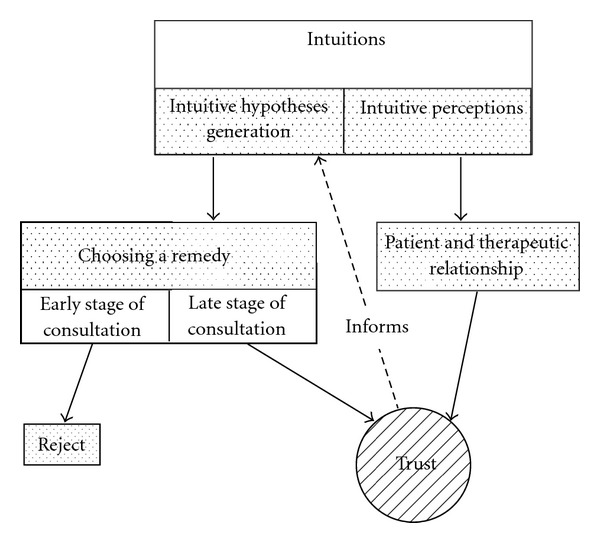
Intuition in homeopathic decision making.

Intuition was a difficult aspect of practice for these practitioners to explicitly define. Four themes are presented that help to uncover its nature.  Figure 1 demonstrates how intuition is used in clinical homeopathic practice.

**Theme 1:**

*Intuition—How Homeopaths Recognize and Describe Intuition.* Participants reported three sub-themes explaining what intuition was to them and how they experienced it (Table 1).

- *Intuition as “a feeling”*. Practitioners often referred to how intuition manifested itself as various feelings, such as, “an inkling”, “a gut-feeling”, “a sense” and “a picture”. Although such descriptions differed across accounts, the form of an intuitive feeling was recognizable to the practitioners; each practitioner could identify within themselves what intuition felt like.
- *Awareness of intuition*. Participants also described different experiences of how aware or conscious they were in terms of experiencing their intuition. Such descriptions from the participants implied a well-developed ability to read people's non-verbal behavior which was consequently interpreted into case information. This was demonstrated on occasions whereby practitioners perceived that there was more underlying what was being said by their patient. The intuitive perception appeared to be an involuntarily arising feeling or acknowledgment concerning a patient. This highlights the notion that intuition is not consciously directed by the practitioner, but rather they receive and understand useful information automatically. This reflects what could be interpreted as gestalt intuition; using perceptual information to judge gaps, missing pieces or hidden relationships between pieces of information. It also resulted in some participants intuitively perceiving the correct remedy for their patient.
- *The rapid manifestation of intuition*. The descriptions of intuition not only showed that the homeopathic practitioner is aware that they are using intuition but also reflected the type of intuition known as cognitive inference whereby the perception of relevant information occurred so rapidly that it was hard to pinpoint exactly what it was that lead to a judgment or intuitive thought.

**Theme 2:**

* Beliefs about the Origins of Intuition.* The majority of the participants felt that their intuitions arose mainly from cognitive sources,that is, knowledge and experience (both clinical and personal) (Table 2). Most participants clearly maintained that their intuitions were rooted within their homeopathic knowledge learnt through their education in homeopathy. The participants also reflected that their intuitions were based on their clinical experience which in turn also contributed to their knowledge, being manifested as embodied knowledge within the practitioner from which further intuitions could also arise. Others also described their intuition as their natural ability to be intuitive. Knowledge and personal intuitive sense were perceived to be intertwined. These aspects emphasize the cognitive elements of intuition; however, one participant also reflected an alternative non-cognitive based rationale for their intuitions. This participant believed that intuition was based on future, not past or present, knowledge and arose from a power outside him; he described these intuitions as being from “higher realms”. This has previously been described in the literature as a pre-cognitive form of thinking.

**Theme 3:**

*Types of Intuition.* Two types of intuition experienced by the homeopaths were identified which demonstrated how intuitions had a specific role during consultations (Table 3).

- *Intuitive perceptions.* All participants considered that some perceptions about their patients were based on intuition. When engaging with patients in the consultation participants would describe how they would pay close attention to the patient, and pick up cues from verbal and non-verbal behavior. These “intuitive perceptions” allowed participants to develop their problem-solving cognitions and were often associated with a deeper understanding of their patient, perhaps allowing a more perceptive analysis.

(b) *Intuitive hypothesis generation*. Participants also described how intuition manifested itself through the sudden onset of idea generation which led them to subsequently engage in a deductive process to test out their hypothesis or idea that would ultimately help them in their decision making. Their testing of the “intuitive hypotheses” indicates a lack of trust of the intuitive thoughts.

**Theme 4:**

*The Selective Use of Intuition: Trusting Intuition.* Participants tended to only use their intuition when they felt it was valid and therefore trustworthy. The participants described intuitions that would enhance two aspects of the consultation process (Table 4).

- *Intuitions about patients and the therapeutic relationship*. Participants reported that some of their intuitions enabled them a more detailed understanding of the patient and hence developed their patient practitioner relationship. These patient-based intuitions were trusted at all stages of the lengthy consultation process. For example, intuitions that led to asking certain questions or delving for more information were trusted and used by the practitioner.

(b) *Intuitions about the remedy ideas and prescription.* In contrast, trust in intuitions for remedy ideas and prescribing decisions depended on the timing of when they occurred in the consultation, and reflects their need to deductively test out their intuitive thoughts because of the possible negative impact for the patient if they got this wrong. Remedy prescriptions would automatically “pop” into their mind during the early stages of the consultation but generally these were not trusted or acted upon although they were noted. They were resistant to these early remedy intuitions since they did not have the full knowledge to make valid prescribing decisions. For most practitioners, without reliable and quantifiable information to back up intuitive feelings there was a definite resistance in relying on them; they inferred that some instances of these early intuitions were outside of the strict and logical rules of the homeopathic prescribing process.

Intuitive feelings for the prescription towards the end of the consultation were viewed with more trust as by this point in the consultation the participants had been through logical and hypothetical deductive processes which narrowed the choice to a few remedies. Here intuition was sometimes used as a tool to make their final prescribing decision helping to give it credibility. The practitioners judged this final decision to be sourced from case information and knowledge rather than biased feelings and reflected a sentiment described by others that homeopathy should be viewed as being logical and knowledge based.

### 4. Discussion

In this first qualitative study assessing intuition in homeopathic practitioners, a number of themes emerged inductively from the transcripts and as data saturation was achieved, we consider the findings robust. The participants interviewed were all experienced practitioners so were more likely than novices to use intuition in their daily practice [1]. Our findings confirm that despite differences in clinical practice, homeopaths show considerable similarities to other medical practitioners in their description and use of intuition in practice. They were aware of experiencing intuition and the rapid nature of the experience [41, 42]; they found it difficult to express what intuition is (e.g., [6, 16]); but had an understanding of its origins during clinical practice, perceiving it to be a cognitive process reliant on their clinical knowledge and experience [43–45]; but also related to their inherent personal intuitiveness [46]. In addition, they were also cognizant of the types of intuition they used; intuitions helping them to deepen their understanding of their patient (perceptive intuition [46]) and enhance their relationship with their patients, as well as those helping their prescribing decisions (both perceptive but also idea generating intuitions). These specific uses reflect, in part, the different distinctions in intuitions described previously by others (e.g., [1, 20]).

Finally, our findings also clearly demonstrate how selective homeopaths are about deciding whether to act on their intuitive thoughts and how their response is dependent upon the nature and timing of their intuitions. Intuitive perceptions which related to understanding the patient and developing the patient-practitioner relationship were always considered valid. This strong acceptance of inter-personal intuitions, although recognized [47], have not been explicitly reported in any previous literature so it is unclear if this is similar or different to other clinicians. It is conceivable that these types of intuitive perceptions may be more emphasized in homeopathic practitioners because of their training and nature of their clinical practice, their holistic approach and lengthy nature of their consultations [48]; knowing the patient does, unsurprisingly, affect decision making [49]. Alternatively, it may solely reflect that this group of experienced clinicians became more perceptive with increasing clinical experience and were more able to pick up “odd things” that informed their intuitions [10]. In contrast, the validity of homeopath's prescribing intuitions (i.e., selecting one remedy out of the possible four thousand remedies) were always interpreted carefully as the consequences could cause risk to their patient if the prescription was wrong. These types of intuitive thoughts had to be verified via hypothetico-deductive reasoning to see if their logical decision matched their intuitive suggestion, hence the importance of the timing of these intuitions during the consultation. This finding reflects previous work in nursing populations and other health care professionals [7, 17, 24, 42, 50]. In nursing populations, a four step behavioral response is used to decide whether to act on intuition [17]. Like nurses, the homeopaths, too, needed corroboration and validation of their intuitions by seeking additional data via focused questions before prescribing the remedy.

The participants in this study all emphatically reported how their final treatment decision was based on following logical processes to identify the correct prescription for their patients. This mistrust of intuition relating to the prescription may in part also relate to personal [51] and societal beliefs, as well as being reflective of the current academic and medical mistrust of both intuition [52, 53] and homeopathy [39, 54, 55] which was alluded to by some homeopaths in this study. Underlying this is the central argument of whether intuition is a rational or irrational phenomenon. The current emphasis on evidence based medicine in clinical practice [56, 57] is at odds with intuition being rational process and may explain the homeopaths' emphatic vocalization that their decision making process is firmly rooted in knowledge and logic.

### 5. Strengths and Limitations

The strengths of this study include a large sample allowing data saturation to be achieved, and in addition, the findings are more transferable as they are based on practitioners across different geographical and socioeconomic areas. All participants were also interviewed by the same researcher and to help ensure a reflexive approach, the analysis was carried out by three researchers. A concern of this study relates to a possible response bias of participants when faced with questioning concerning intuition. Participants may have provided answers and explanations that are non-controversial since the findings will be published in academic medical literature.

### 6. How Does This Further Our Understanding of Intuition?

Homeopaths share many of the similarities to other health professions about how intuition is viewed, manifested and used in practice. Like other clinicians, intuition relating to treatment interventions was not trusted implicitly but instead tested out deductively. This testing out may be aided by the length of their consultations, their training, but, also may reflect their perceptions from academia and the media regarding the credibility of homeopathy. This data may therefore provide these practitioners legitimacy of using intuition during decision making. On the other hand, their acceptance of intuitions relating to either the patient and/or their relationship was striking. It is feasible that this may relate to the nature and length of their specific type of consultation; and further work would elucidate this.

### 7. Where Do We Go from Here?

Since intuition is one of a number of tools used in clinical decision making, its recognition and its contextual use is relevant having implications for the training of these and other healthcare practitioners. Various techniques such as creative writing, narrative work and group discussion methods [58] could be employed to help develop and refine the practitioner's intuitive skills. Further research in other forms of private and NHS holistic style and/or complementary medical therapy consultations should be explored to identify if these findings are consistent with this group of practitioners. In addition, further exploration of medical homeopaths and their perception of intuition would be enlightening as those working in the NHS have less consultation time than private homeopaths and may use intuition in a different way.

### Funding

National Institute of Health Research (PDA04/CAMs2/02 to S.B.) and The Wellcome Trust Biomedical Vacation Scholarship Fund (VS/06/SOUTH/A3 to A.B.).

## Figures and Tables

**Table 1:** How homeopaths recognize and describe intuition.

Subtheme	Example
Descriptions of intuitions	Intuition is more of a felt sense … more a sort of gut-feeling.” “The picture will come to me; a wholeness will come to me. And that's intuition … it's almost as though it's a kind of visual felt sense.” “That's another thing that you get an inkling about, or I get an inkling about, is how much I can question someone … so I'm quite sensitive about it … I don't usually get that wrong really, usually I know how far I can go with someone.”
Recognizing intuition (a) Awareness of intuition depends on the practitioner's level of sensitivity	“I am very conscious of it because I'm using it as a tool to understand what's going on. I think that it's a level of sensitivity … it's something that everybody has … we all have gut-feelings, inklings, call it intuition, and I think what that is based on is experience and knowledge … I think that often people will have a kind of semi-awareness of it, some people are very aware of it, some people are very unaware of it. […] you know it's quite a heightened state of being focused on what someone's saying, you kind of train yourself to be even more aware so it's like you're in a higher level on sensitivity.”
(b) Based on interpreting non verbal behavior: reading between the lines	“You get a kind of inkling that something, you know there is more to just the bladder problem from the way that she's talking and the things that she mentions, maybe her body language.”
(c) Based on a feeling—gestalt intuition	“Intuition is that sense that you know what remedy someone needs.”
Happens very rapidly: cognitive inference	“Something comes in when you're with a patient, something comes in to your head, like a little light I suppose, something tells you they might need that remedy.”

**Table 2:** Beliefs about the origins of intuition.

Subtheme: based on	Example
Knowledge	“Intuition is … based on knowledge that you've learned in a structured way.”
Clinical experience	“There must always be an element of art, intuition … past experience … I now have 20 yrs experience of how remedies work on people, what sort of people can benefit from a particular remedy.”
Embodied knowledge (knowledge and clinical experience)	“I do think it's what I've said, experience and knowledge, and I think listening to your inner processes about that.”
Personal *experience (intrinsic intuition)*	“Well the word “intuition” means self knowledge I guess. It's largely based on your own experiences, it's in you and it's what you've learned. Based on […] experiences that you may or may not have fully understood at the time.”
Unconscious merging of knowledge and personal experience	“Well basically intuition arises because you've got knowledge yourself … Intuition is the marriage of those two things; their information and my intrinsic knowledge, not conscious. Intuition is the unconscious realization of something that arises out of that. You don't have intuition if you don't have knowledge. If I don't know a remedy, I am not going to “intuit” it.”

**Table 3:** Types of intuition.

Subtheme	Example
Intuitive perceptions	“I can base some of my questioning … the way that I move into somebody through intuition … having a sense of the individual's own energy. And all of these things come into it, it's not only the words they say but the way their voice is.”
Intuitive hypothesis generation	“You'll have a set of circumstances, a set of conditions that a person has got, and then all of a sudden you'll say, and you don't know why you're asking it, “have you ever had a head injury?” … and then they'll say, “oh well I fell off a horse” … and then you think, that's it.”

**Table 4:** The selective use of intuition: trusting intuition.

Sub-theme	Example
Intuitions about patients and the therapeutic relationship	“I think my intuition comes at the level of personal contact. So that I will know when to prod and when to explore.”

*Intuition about remedy ideas during the early stages*	

Noting them …	“I might jot it down, in the margin. At that point that was the remedy I thought of. Then they'll move onto something else … so then I start to ask a few questions around the remedy that I'm thinking of, just to see.”
But being wary of them	you've still got to have what the patient will tell you, and if they miss the big point or you miss the big point, you will not help them. Because the intuition (about the remedy) will only work on the information it receives, won't it?”
	“(if) someone just sits down and bursts into tears, you think Ignatia … if I'm thinking of a remedy quite early in the consultation, it makes me aware that I've got to put that out of my mind so that I'm not prejudiced.”
Because they need to be verified through case taking	“I think intuition plays a massive role in it but you try and stick to being non-judgemental … you can't bring your views and opinions, you have to verify if you've got a feeling, a gut-feeling or a cerebral feeling … you have to be sure about that, so I need to quantify it.”

*Intuition about remedy ideas during the latter stages*	

As a tool to make their final decision helping to give it credibility	“I think the danger of me emphasising the intuitive bit is that, I'm being honest with you that I use that, but one must be aware that one's doing that, and not say, right I've made my decision in the first ten minutes. You've really got to say, okay and shove that to the back of your mind. We'll go through the routine and we'll see if we still come out to the conclusion at the end … It would be wrong to say that's not at the back of my mind and I'm checking it and I'm making sure that I have got the right feeling because if distinctly in the first twenty minutes I was thinking, oh no I'm completely in the wrong ballpark, then I can put that to the back of my mind and say, no let's hear the real story … because ideally I think you would go into it as a completely blank slate. I think in all honesty we all have feelings and intuitions, and you can't help but not.”
But may guide the final decision	“In the final decision there will come into it some notion of feelings … this is the art really.”
Overarching concern: homeopaths want their decisions to be viewed as being logical and knowledge based.	“You get an understanding or a feeling for that remedy, and that takes you away from the complete logic which I really like. There is this other aspect of what feels right and what doesn't feel right … I feel slightly uncomfortable talking about that because I feel that homeopathy needs to be seen as something that has a basis in science and in logic and in principles.”
